# Trans-cranial Doppler as an Ancillary Study Supporting Irreversible Brain Injury in a Post Cardiac Arrest Patient on Extracorporeal Membrane Oxygenation

**DOI:** 10.7759/cureus.2161

**Published:** 2018-02-06

**Authors:** Naresh Mullaguri, Aarti Sarwal, Nakul Katyal, Premkumar Nattanamai, Pravin George, Christopher R Newey

**Affiliations:** 1 Neurology, Cleveland Clinic Ohio; 2 Neurology, Wake Forest School of Medicine; 3 Neurology, University of Missouri Columbia

**Keywords:** brain death, extracorporeal membrane oxygenation (ecmo), computed tomography (ct), transcranial doppler (tcd)

## Abstract

Obtaining neuroimaging in patients on cardiopulmonary support devices such as extracorporeal membrane oxygenation (ECMO) can be challenging, given the complexities in monitoring, instrumentation, and associated hemodynamic lability. Transcranial Doppler (TCD) is used as an ancillary test for the assessment of cerebral circulatory arrest, but its use in non-pulsatile blood flow in venoarterial (VA) ECMO is not well described. We report the use of TCD in a patient on VA ECMO post-cardiac arrest for evaluation of death by neurological criteria. A 72-year-old female was admitted for elective trans-catheter aortic valve replacement. Her postoperative course was complicated by hemo-pericardium evolving into pulseless electrical activity causing cardiac arrest. She was resuscitated with return of spontaneous circulation and initiated on VA ECMO and intra-aortic balloon pump for cardiogenic shock. Over the next few days, serial evaluations persistently showed a poor neurological examination. She was too unstable to transport for neuroimaging. Evaluation for death by neurological criteria was performed with a clinical examination, apnea testing, and TCD as an ancillary study. TCD showed systolic spikes supporting an impression of cerebral circulatory arrest consistent with an irreversible brain injury.

## Introduction

Presence of irreversible brain injury on neuroimaging is an essential pre-requisite of determining death by neurological criteria [1]. Unfortunately, obtaining neuroimaging, such as a computed tomography (CT) head, safely may not be possible in select subset of patients due to hemodynamic instability or instrumentation impairing safe transport, such as those patients on venoarterial extracorporeal membrane oxygenation (VA ECMO). In these patients, suspicion of brain death in a patient where physiology and extent of brain injury may not be fully understood must be confirmed with diligent serial clinical examination, thorough apnea testing, and ancillary testing. Guidance on determining brain death in this patient population is guided largely by case reports and case series [2-4]. Currently, there are no guidelines for ancillary testing for assessing cerebral circulatory arrest for evaluation of death by neurological criteria in patients on VA ECMO. We report the use of transcranial Doppler (TCD) as ancillary testing in a patient on VA ECMO who was too unstable for transportation to neuroimaging. We used a combination of clinical history, neurological examination, apnea testing, and bedside TCD to demonstrate an irreversible brain injury leading to a mechanism for brain death.

## Case presentation

**Figure 1:**
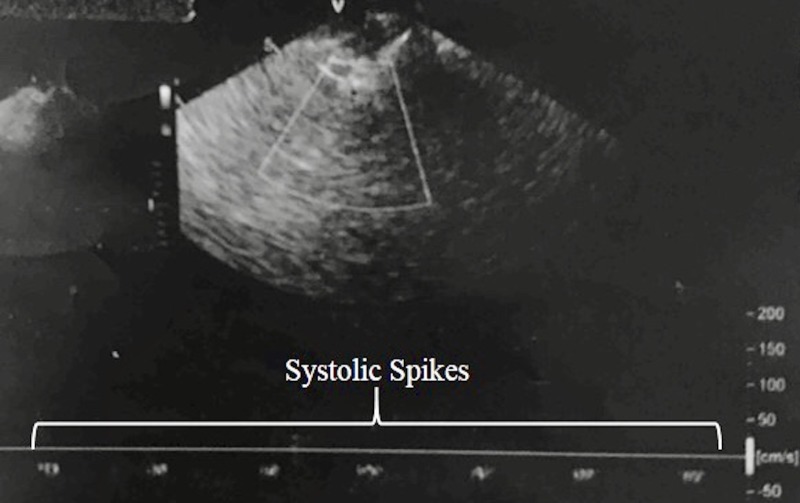
Transcranial Doppler (TCD). TCD showing systolic spikes in the anterior circulation.

A 72-year-old lady was admitted for elective trans-catheter aortic valve replacement (TAVR). Her postoperative course was complicated by hemopericardium that progressed to pulseless electrical activity. As part of resuscitation, she underwent emergent pericardiocentesis and was placed on VA ECMO and intra-aortic balloon pump (IABP) for cardiogenic shock. Over the following two days, she was recognized by the primary cardiology service to have a poor neurological examination including nonreactive pupils and not triggering the ventilator. Her underlying heart rhythm remained pulseless electrical activity. Neurology was consulted for prognostication after presumed anoxic brain injury. Initial evaluation was performed off sedation at body temperature of 36.4C. Mean arterial pressure was sustained above 70 mmHg by VA ECMO, IABP, and norepinephrine with no severe electrolyte or metabolic derangement. There were no fluctuations in heart rate with noxious stimulation. Her pupils were 4 mm and nonreactive bilaterally. She had no corneal reflex. Her eyes remained midline during oculocephalic and oculovestibular testing. She had no cough or gag. She had no evidence of spontaneously initiated breaths. All four limbs were atonic with no withdrawal to painful stimuli. Deep tendon reflexes and plantar responses were absent bilaterally. Given the complexity of cardiopulmonary instrumentation and monitoring, CT head could not be safely obtained. We used bedside TCD to evaluate for cerebral circulatory arrest to support the clinical impression of an irreversible brain injury. The TCD and repeated TCD showed isolated systolic spikes in the anterior and posterior circulation in all insonated vessels consistent with cerebral circulatory arrest (Figure 1).

Reversal of systolic spikes in the middle cerebral artery was likely oscillatory waveform from the artificial circulatory support provided by the combination of VA ECMO and IABP. After the clinical examination and TCD, apnea testing was then performed. She was on pressure control mechanical ventilation. The Fraction of inspired oxygen (FiO_2_) was increased to 100% to preoxygenate. Arterial blood gases (ABG) showed PaO_2_ of 236 mm of Hg and PaCO_2_ of 39 mm of Hg. She was then disconnected from the ventilator. A cannula with 4 liters of O_2_ flow was placed inside the endotracheal tube at the level of the carina. The sweep rate on the VA ECMO was concomitantly reduced to 800cc and monitored with arterial line and pulse oximetry. O_2_ saturation was sustained at 96 to 100% throughout apnea testing with no change in systolic blood pressure which was supported with vasopressors, VA ECMO, and IABP. After approximately 12 minutes, ABG showed PaCO_2_ of 68 mm of Hg. She was declared brain dead.

## Discussion

Evidence of irreversible brain injury on neuroimaging is an essential pre-requisite to diagnosing death by neurological criteria [1, 5]. Typically neuroimaging is obtained to support the clinical impression of irreversibility of brain injury since clinical neurological examination and apnea testing can be confounded by many medical and pharmacological factors [6]. Additional ancillary testing may be necessary in certain circumstances when multiple confounders are present. These ancillary tests include either demonstration of absent electrical activity by electroencephalography (EEG) or evidence of cerebral circulatory arrest by imaging modalities such as cerebral angiography, nuclear single-photon emission computed tomography (SPECT) scan, or TCD [1,6].

Patients on assistive cardiopulmonary support, such as ECMO or IABP, have a high incidence of neurological complications as well as high incidence of severe brain injury [7-9]. Necropsy studies on ECMO patients showed significant hypoxic ischemic changes in vulnerable cortical, subcortical, and cerebellar regions possibly due to hypoperfusion and/or hypoxemia along with coagulopathy causing intracerebral and/or subarachnoid hemorrhages. Early recognition of severe brain injury especially when it portends possible irreversibility is quite pertinent in such patients. Neuroimaging has an important role for this evaluation but obtaining neuroimaging, such as CT head, in these patients can be difficult due to instrumentation, complexity of monitoring, and hemodynamic lability. This difficulty was highlighted by the case series by Mateen, et al. In this study, the majority of patients could not be imaged. Only 24 out of 87 patients on VA ECMO had neuroimaging. When able to be imaged, the majority of these patients showed abnormal findings on their neuroimaging with a high incidence of progression to brain death. Bedside ancillary tests may be especially helpful in patients on ECMO, where apnea test can be difficult to perform and neuroimaging can be difficult to obtain. Review of literature regarding evaluation of patients for death by neurological criteria revealed variable practices on the use of neuroimaging/ancillary testing in patients on ECMO [7]. Some reports declared patients using only clinical examination and apnea testing [4]. The use of the bedside EEG or TCD may be useful in supporting the clinical impression of an irreversible brain injury in patients who are too unstable to transport to neuroimaging as we have reported. Use of TCD is challenging in VA ECMO due to the non-pulsatile nature of blood flow created by assistive cardiopulmonary bypass. Presence of significant embolic signals from the continuous flow of blood through an external cardiopulmonary bypass also needs consideration. Previous studies have shown feasibility of assessing cerebral blood flow in patients on VA ECMO using TCD. Since blood flow can be discerned with certain level of confidence using TCDs in VA ECMO, criteria of systolic spikes and oscillating waveforms can be reliably used to evaluate cerebral circulatory arrest when systemic blood pressure is maintained [10].

Our case highlights the challenge of determining an irreversible brain injury without neuroimaging. The use of TCD at bedside to determine cerebral circulatory arrest along with the clinical history and examination provided confidence necessary to determine irreversibility of brain injury. Although case reports have been published describing brain death testing on patients requiring VA ECMO, none have addressed the complexity of obtaining neuroimaging and provided guidance on bedside testing. It will be extremely helpful to gather more data and create practice guidelines on the use of TCD to assess cerebral blood flow and evaluation of brain death determination in patients on ECMO. Additionally, guidelines will need to take into consideration on how to define irreversible brain injury when neuroimaging cannot be obtained.

## Conclusions

In patients on VA ECMO, ancillary testing using TCDs can be a reliable neuroimaging assessment to determine irreversible brain injury to supplement the clinical history and clinical examination suspicious for death by neurological criteria. Future guidelines will need to provide guidance on defining an irreversible brain injury when unable to obtain neuroimaging.

## References

[1] Wijdicks EF, Varelas PN, Gronseth GS (2010). Evidence-based guideline update: determining brain death in adults: report of the Quality Standards Subcommittee of the American Academy of Neurology. Neurology.

[2] Giani M, Scaravilli V, Colombo SM (2016). Apnea test during brain death assessment in mechanically ventilated and ECMO patients. Intensive Care Med.

[3] Hoskote SS, Fugate JE, Wijdicks EF (2014). Performance of an apnea test for brain death determination in a patient receiving venoarterial extracorporeal membrane oxygenation. J Cardiothorac Vasc Anesth.

[4] Saucha W, Sołek-Pastuszka J, Bohatyrewicz R (2015). Apnea test in the determination of brain death in patients treated with extracorporeal membrane oxygenation (ECMO). Anaesthesiol Intensive Ther.

[5] Wijdicks EF (2015). Brain death guidelines explained. Semin Neurol.

[6] Van der Lugt A (2010). Imaging tests in determination of brain death. Neuroradiology.

[7] Mateen FJ, Muralidharan R, Shinohara RT (2011). Neurological injury in adults treated with extracorporeal membrane oxygenation. Arch Neurol.

[8] Ryu JA, Cho YH, Sung K (2015). Predictors of neurological outcomes after successful extracorporeal cardiopulmonary resuscitation. BMC Anesthesiol.

[9] Martucci G, Lo Re V, Arcadipane A (2016). Neurological injuries and extracorporeal membrane oxygenation: the challenge of the new ECMO era. Neurol Sci.

[10] Baghshomali S, Reynolds P, Sarwal A (2014). Transcranial Doppler to assess cerebral blood flow in patients on extra corporeal membrane oxygenation. Neurology.

